# Biocircularity: a Framework to Define Sustainable, Circular Bioeconomy

**DOI:** 10.1007/s43615-022-00180-y

**Published:** 2022-06-08

**Authors:** Nicholas M. Holden, Andrew M. Neill, Jane C. Stout, Derek O’Brien, Michael A. Morris

**Affiliations:** 1 School of Biosystems and Food Engineering, University College Dublin, Dublin, Ireland; 2 BiOrbic Bioeconomy, SFI Research Centre, University College Dublin, Dublin, Ireland; 3 Botany Department, School of Natural Sciences, Trinity College Dublin, Dublin, Ireland; 4 School of Chemistry, Trinity College Dublin, Dublin, Ireland; 5 Amber, SFI Research Centre, Trinity College Dublin, Dublin, Ireland

**Keywords:** Biological materials, Feedstock, Extended life, Natural capital, Life cycle assessment, Transition

## Abstract

Bioeconomy is proposed as a solution to reduce reliance on fossil resources. However, bioeconomy is not always inherently circular and can mimic the conventional take, make, consume, dispose linear economic model. Agricultural systems will be relied on to provide food, materials, and energy, so unless action is taken, demand for land will inevitably exceed supply. Bioeconomy will have to embrace circularity to enable production of renewable feedstocks in terms of both biomass yield and maintaining essential natural capital. The concept of biocircularity is proposed as an integrated systems approach to the sustainable production of renewable biological materials focusing on extended use, maximum reuse, recycling, and design for degradation from polymers to monomers, while avoiding the “failure” of end of life and minimizing energy demand and waste. Challenges are discussed including sustainable production and consumption; quantifying externalities; decoupling economic growth from depletion; valuing natural ecosystems; design across scales; renewable energy provision; barriers to adoption; and integration with food systems. Biocircularity offers a theoretical basis and measures of success, for implementing sustainable circular bioeconomy.

## Introduction

Current economic models and a “business-as-usual” approach have become incompatible with a prosperous, safe future for humanity [1]. Modern society is reliant on fossil fuels for food, industry, and infrastructure, with most societies relying on economic growth driven by a take, make, consume, dispose model. The bioeconomy has been defined by the European Union as “…all sectors and systems that rely on biological resources…, their functions and principles [including] land and marine ecosystems and the services they provide…all economic and industrial sectors that use biological resources and processes…” [2]. However, early bioeconomy innovations replaced oil- or mineral-derived materials with biologically derived materials [3], introduced valorization of waste streams [4], and bioenergy [5]. All potentially lead to increased exploitation of already unsustainably utilized natural resources [6]. Under these circumstances, demand for products places a proportional pressure on natural resources, even if the whole process becomes more eco-efficient (i.e., less impact per unit output) [7]. It is necessary to break the link between economic growth and resource depletion [8] and to recognize the biosphere does more than simply provide physical commodities. Ecosystems provide a suite of benefits, but only a subset of their value is captured by the conventional economic system, the remainder being described as “silent” and “invisible” [9]. Further damage to ecosystems will have severe economic impacts [10].

Sustainable bioeconomy requires renewable resources that will remain available on an ongoing basis. For biological resources, mere stock replacement has been interpreted as renewable feedstock, with actors simply assuming crops are renewable [11], when it is the stock of underpinning natural capital that should be considered [12]. The replacement rate for a given biomass resource is dependent on the underpinning stock of natural capital. If the necessary natural capital assets are lost or degraded or biomass extraction exceeds the rate of replacement, long-term ecosystem service flow will be compromised. Under these conditions, a feedstock is not sustainable and should not be considered renewable, regardless of biological regeneration time. A critical question for bioeconomy is how much bioresource can be harnessed or alternatively the maximum ecosystem service flow that can be realized, without impeding long-term regeneration and without decreasing other irreplaceable ecosystem services? The bioeconomy is as vulnerable as other economic systems to perpetuating common failures of the past century, particularly increasing the flow of material services from the biosphere to society at the expense of regulating, maintenance, and cultural services [11], at a global level.

Circular economy should decouple economic growth from resource use by lifetime extension, increased use intensity (e.g., by longevity, sharing and rental), reuse, repair, reverse logistics, remanufacture, recycling, and valorization whilst maintaining value, thereby minimizing waste and promoting regeneration [13]. This means that to keep pace with increasing demand due to population growth and lifestyle patterns, humans should use fewer virgin materials because more materials are kept in circulation and less are wasted [14]. Circular economy should also support the regeneration of natural systems to undo the harm of the last two centuries [15], but this has not been a focus of academic inquiry [16].

Circular bioeconomy “…focus[es] on the sustainable, resource-efficient valorization of biomass in integrated, multi-output production chains (e.g., biorefineries) while also making use of residues and wastes and optimizing the value of biomass over time via cascading…” [17]. Theoretically, circular bioeconomy is an approach that can provide society with a sustainable future by allowing economic growth within the planetary boundaries and decoupling demand from resource extraction, but fears have been expressed that it will increase pressure on natural ecosystems [18]. To make sustainable circular bioeconomy a reality, a deeper understanding of what it really means must be developed among all stakeholders. The International Standards Organization Technical Committee 323 (ISO/TC323 [19]) on standardization in the field of circular economy will provide some agreed definitions to aid understanding. However, many of the concepts of circular and bio-economy overlap and no single approach can work everywhere, for everyone [20]. This means time- and site-specific solutions will need to be designed to fit each circumstance and exceed current visions of the bioeconomy [21].

The only way to achieve a truly sustainable, circular bioeconomy is to embrace all elements of circularity including eco-design of products, use of processes, and services that drive integrated systemic thinking, targeting sustainable production and consumption of renewable biological materials and prioritizing maintenance and enhancement of natural capital. It is necessary to move from a theoretical combining of bioeconomy and circular economy to a framework of concepts and metrics that allow stakeholders to plan for sustainable circular bioeconomy. This theoretical work develops from recent discussion of circular bioeconomy, considering opportunities and limitations [22], its role [17], and drivers [23]. A means of knowing when a material or product is contributing to sustainable, circular bioeconomy is required. A formal conceptual framework, called *biocircularity*, is outlined that defines fundamental attributes of the continuum from feedstock through material to objects (i.e., useful products) for sustainable, circular bioeconomy and provides an unambiguous framework against which all actors and stakeholders can plan and implement sustainable, circular bioeconomy developments. The term biocircularity is not intended as an alternative descriptor of sustainable circular bioeconomy, but as a means of assessing when a system or technology change or innovation will contribute towards decoupling use from resource extraction and its impacts. Important challenges to be overcome to develop a holistic understanding of a mature economy that meets the needs of society and the planet are discussed.

## The Concept of Biocircularity

**Fig. 1:**
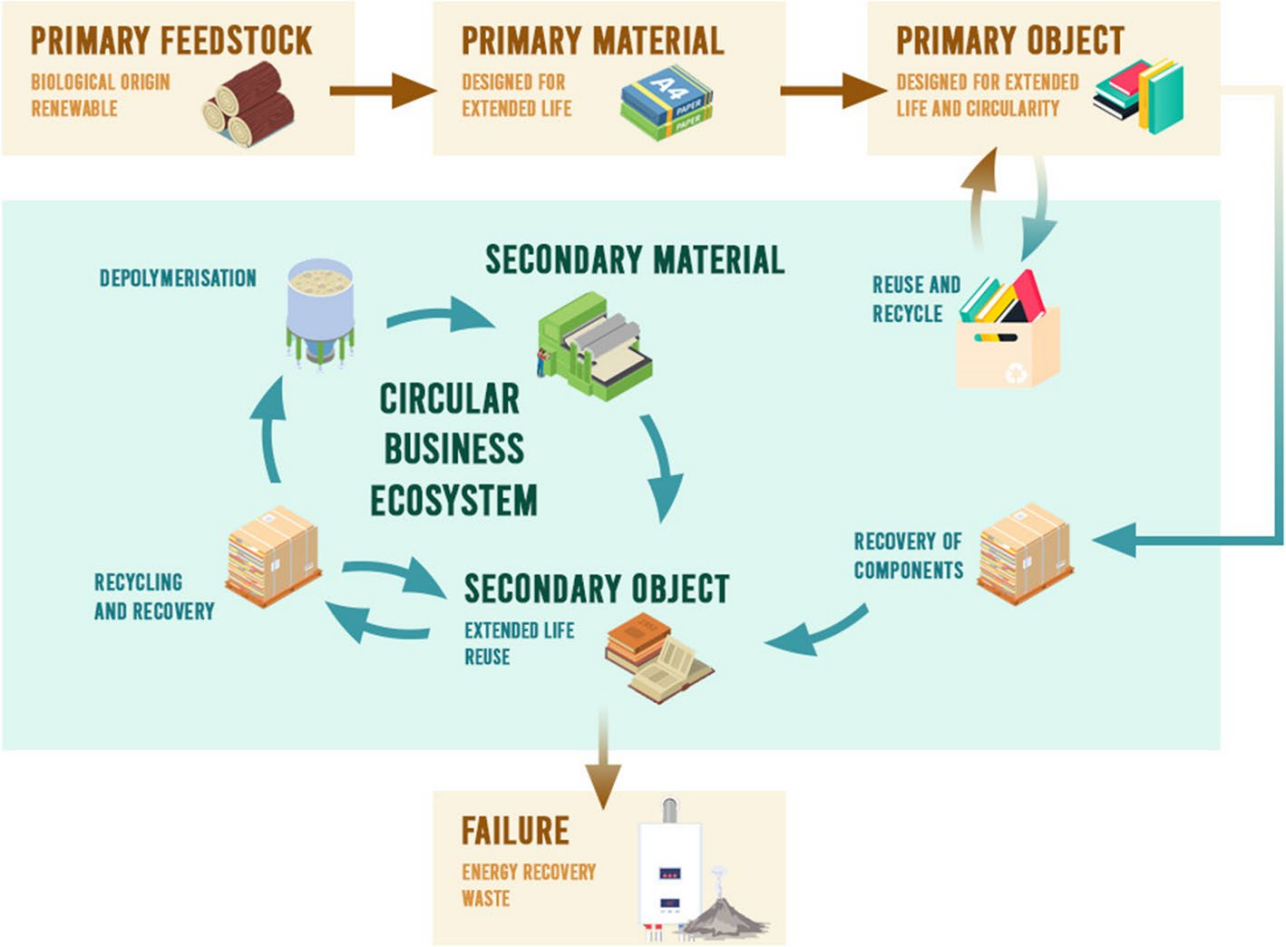
The relationships between feedstock, material, and objects in a circular bioeconomy (source: the authors)

The concept of biocircularity is proposed to provide an unambiguous framework against which all actors and stakeholders can plan and implement circular bioeconomy innovations for materials and products. The relationships between stages in a circular bioeconomy are outlined in Fig. 1. Biocircularity has six quantifiable attributes that can be defined using unambiguous criteria (Table 1):

1. The primary feedstock (also referred to as a resource) is of biological origin but is not extracted from a carbon sink such as coal or peat that has been subject to short- or long-term physical transformation. The primary feedstock should be organic matter comprised of recently living cells or a chemical substance emitted from a living organism. This is a classifiable attribute of the system that can be recorded using standard stock inventory and flow methods. The geographical location of feedstocks, as well as extent and condition of natural capital stocks, should also be recorded to account for spatial variation in natural capital (stocks, condition, and flows).
2. The primary feedstock is renewable and should not deplete natural capital where it is produced. Usable metrics include land use, water use, and greenhouse gas emissions [24], but a much wider range is available [20]. Many of these can be measured using life cycle assessment methods [25] or earth system models [26]. There is ongoing development of natural capital accounting methods [27] that could also be applied to measure this attribute as well as methods such as ecological footprint [28], but this has some serious shortcomings for use in the context of biocircularity [29].
3. Materials (derived from primary resources, matter from which objects are made) are designed for extended life. Materials (monomers, polymers, composites) produced from the feedstock should be designed to be suitable for objects that can have extended use and reuse before becoming too degraded for their primary purpose (the green cycle in Fig. 1). The material degradation time can be used to determine if it will minimize pressure on primary feedstocks to allow low impact, renewable management. The greater the demand for a material, the longer it will have to remain in an (almost) closed cycle of transformations to minimize adverse impacts on natural capital from excessive extraction. Mass flow analysis methods have already been shown to be suitable for this type of measurement [30]. Furthermore, the technology used to make the primary materials from feedstocks should enable maximum valorization of all component parts of the feedstock. The goal should be zero resource loss, which is measurable using mass balance methods. As the circle is closed, primary materials will increasingly be replaced by secondary materials recovered from existing objects (Fig. 1). In practice, a perfect system is unlikely to be possible, so important metrics describing resource use efficiency will be required, both for specific feedstock/material combinations [31] and at the corporate level [32].
4. Objects (useful products) are designed for extended life and circularity. Objects made from the material(s) should be designed to maximize (a) lifetime; (b) reuse; (c) repair; (d) remanufacture; (e) recovery of components; (f) recycling; (g) useful resource extraction; and finally (h) energy extraction. The processing system that creates objects must be designed to do more than merely cascade the biomass [17]; rather, it must minimize energy demand, losses, emissions, material degradation, mass loss during use cycles, and minimum loss/energy demand during the transition from one material/product to the next. This means that a feedstock and the primary materials must be developed using a holistic design that considers the primary use of the object and the secondary use of the materials from which it is made. There are now a wide range of “design for sustainability” methods proposed [33] and energy and material flow analysis tools [34] that can be used. All have shortcomings, but these design methods and measurement methods (such as LCA) should be used to understand and measure this attribute. Several index methods such as repairability [35, 36], recyclability [37], and recovery could be further developed. There is a need for transdisciplinary research to develop holistic design methods and measures of success.
5. A circular business ecosystem of consumers, producers, businesses, and natural resources is required. Subsequent re-processing of components and recycled objects should be part of a designed cascade with planned development of secondary objects (also useful products) and a circularity ecosystem for planned management of recycling and recovery. This must account for the degradation of materials such that depolymerization will be necessary to allow processing to secondary materials. Design of reuse and recycling and recovery should have minimum energy requirements. Theoretically, the difference between market demand and sustainable primary feedstock supply provides an indication of how much mass would need to remain in closed-loop circulation within the economy, which would allow calculation of the time materials should stay in circulation, or the number of cycles required, prior to energy extraction and end of life. While material flow analysis can be used for these calculations, at present methods do not exist to reliably measure this attribute, so research is required, but it is a solvable problem.
6. Failure of the end of life of materials is avoided. The cycling/cascade of the circularity ecosystem should aim to avoid the “failure” of end of life for as long as possible. When degradation has driven materials to be unusable for products or recovery of materials as secondary feedstocks, remaining energy can be extracted, and residue returned to the biosphere sink. In a theoretically perfect system, this step never happens. The gap between the current situation and the ideal can be quantified by measuring, registering, and recording all materials that are incinerated, land-filled, or used for energy supply using material flow analysis [32]. This measurement can be built on existing regulation and monitoring to maintain oversight of end-of-life disposal.

**Table 1:** Unambiguous criteria for defining biocircularity

	Attribute	Criteria/methods
1	The primary feedstock is of biological origin	*The source was derived from a living thing* Determined by meeting seven standard criteria: feeding, movement, respiration, excretion, growth, sensitivity, and reproduction
2	Primary feedstock is renewable	*Regrowth is not at the expense of other ecosystem functions and services* Determined by Natural Capital Accounting or Life Cycle Assessment methods to assess regrowth rates, other biological consequences, adverse environmental impacts, and loss of ecosystem functions. Ideally production of feedstock should be regenerative
3	Materials are designed for extended life	*Materials strength and durability* Determined by materials testing including degradation time, strength, failure conditions, and potential for depolymerization (if appropriate)
4	Objects are designed for extended life and circularity	*The object needs as little energy as possible to disaggregate into components and/or materials* Determined by eco-design guidelines and life cycle assessment. Can also use recovery, recycling and repairability indexes
5	Circular business ecosystem	*More material is in the circular business ecosystem than demand,* and *primary feedstock exploitation rate is orders of magnitude smaller than demand* Determined by material flow analysis and life cycle assessment of products and processes in the context of market data
6	Failure of the end of life of materials is avoided	*Less material is used for energy recovery and routed to disposal than the requirement for primary feedstock* Determined by material flow analysis and market data to assess the amount of material used for energy recovery, directed to incineration and landfill

## The Challenges

To make sustainable, circular bioeconomy a reality that will help countries meet national resource requirements within the ceiling of the planetary boundary, from the foundation of minimum social standards [38] and meet UN Sustainable Development Goal targets (Table 2), biocircularity raises some important challenges. Biocircularity is difficult to achieve because of the need to create value networks and markets into which the feedstock/material/object triple fits and the need to define the space and time over which to measure impact. The key challenges and some suggested solutions are discussed below.

**Table 2:** Some of the relationships between key UN Sustainable Development Goal targets and biocircularity. Other relationships can be identified, but this table focuses on some of the most direct linkages

Key SDG and targets	Observations related to biocircularity
*SDG2: End hunger, achieve food security and improved nutrition and promote sustainable agriculture*	
2.1	A key challenge for biocircularity is to ensure competition between food and bioeconomy does not adversely impact nourishment, hunger, and food security
2.2	
2.3	Bioeconomic production should not mask indicators related to food production. The food first prioritization is required by biocircularity
2.4	
*SDG7: Ensure access to affordable, reliable, sustainable, and modern energy for all*	
7.2.1	While biocircularity defines recovery of energy from materials as a last resort, any bioenergy recovered will be renewable according to the strict definition proposed
*SDG8: Promote sustained, inclusive, and sustainable economic growth, full and productive employment, and decent work for all*	
8.4.1	Biocircularity should aid in promoting global resource efficiency, however, material footprint indicators should explicitly separate bio and fossil materials to better indicate transition to sustainability
8.4.2	
*SDG9: Build resilient infrastructure, promote inclusive and sustainable industrialization and foster innovation*	
All indicators	The indicators used do not distinguish between infrastructure that supports linear, fossil economy and that designed for sustainable circular (bio)economy. Separation of bio and fossil economy and linear and circular infrastructure would be more useful to indicate sustainability. Biocircularity offers the definitions and measurements required to create the necessary datasets
*SDG10: Reduce inequality within and among countries*	
All indicators	Separation of bio and fossil economy and linear and circular infrastructure would be more useful to indicate sustainability. Biocircularity offers the definitions and measurements required to create the necessary datasets
*SDG11: Make cities and human settlements inclusive, safe, resilient, and sustainable*	
11.6.1	A critical factor for the success of sustainable circular bioeconomy will be the infrastructure developed to circular material from urban environments back into the material and object processing cycles. Data on separation, segregation, and direction to reuse and recycling are essential. Biocircularity offers the definitions and measurements required to create the necessary datasets
11.a	
*SDG12: Ensure sustainable consumption and production patterns*	
12.1	National scale action for material and object level circularity management is required. If companies work using biocircularity, 12.1 will be easier to achieve because biocircularity offers the definitions and measurements required to create the necessary datasets
12.2	Material footprint indicators should explicitly separate bio and fossil materials to better indicate transition to sustainability. To support sustainable circular bioeconomy, bio and fossil and linear and circular should be separated into different targets
12.3	Management of food loss and waste will be easier to achieve with biocircularity because it promotes sustainable use of all land and feedstock and material resources in an integrated manner
12.5	Targets prioritize recycling over reuse, use intensification and repurposing, which is contrary to priority of circular bioeconomy. Biocircularity definitions and measurements will make it possible to create the necessary datasets for better indicators
12.6	Companies using biocircularity as a yardstick for measurement and ambition are more likely to have meaningful sustainability reporting
12.8	Biocircularity offers the definitions and measurements required to create the necessary datasets to provide the reliable information needed to achieve the target
*SDG13: Take urgent action to combat climate change and its impacts*	
All indicators	While biocircularity does not prioritize climate impact, it is an integral component of the concept, and a biocircular system should be aligned with all targets, because biomass production depends on a safe and stable environment, including climate, that can support consistent ecosystem service flows over time
*SDG 15: Protect, restore, and promote sustainable use of terrestrial ecosystems, sustainably manage forests, combat desertification, and halt and reverse land degradation and halt biodiversity loss*	
15.1	The use of natural capital concepts to define the sustainability of the feedstock supply directly addresses this target
15.3	Biocircularity requires feedstock to be renewable and ideally regenerative through the maintenance and enhancement of natural capital. Biocircularity should be a driver for meeting this target
15.5	While biocircularity is not concerned with specific species, adopting the principles will reverse habitat loss and reduce pressure on threatened species
15.6	Closely related to the Convention on Biological Diversity (CBD). Biocircularity is expressed in terms of resources, flows, and impacts, but goes hand-in-hand with social equality, ethics and fair access. These issues need to be considered in the future
15.9	Biocircularity definitions and measurements will make it possible to create the necessary datasets for integration of sustainable circular bioeconomy with local and national ecosystem and biodiversity planning and regulation

- *Challenge 1*: *Defining and measuring sustainable production and consumption across all uses of land, sea, and natural capital stocks*. The Sustainable Development Goal 12 (*Ensure sustainable consumption and production patterns*) definitions and indicators represent a political compromise that is not specific enough to meet this challenge. A working definition of a sustainable, circular bioeconomy is an economy that maintains or enhances natural capital while using sustainably produced biological feedstocks to provide materials and services that systemically maintain a circular flow of materials and objects with the intention of avoiding the failure of end of life. The route to a solution for this challenge includes finding the willingness to agree and use the word “sustainable” in an unambiguous way [39] (remove the greenwash and adopting the principles of strong sustainability) and agreeing evaluation methods for circularity and absolute impact. It will be necessary to agree rules and methods to measure the balance of production and consumption through space and time to quantify the gap between the amount of sustainable feedstock (and thus raw materials) available to society and the demand for those materials. This gap is indicative of the amount of material that needs to circulate and the rate of end-of life loss that can be associated with a sustainable, circular bioeconomy.
- *Challenge* 2: *Quantification of externalities and valuation of impacts and natural capital to place ecosystems at the heart of the bioeconomy*. Quantification is necessary to direct and measure transformation from business as usual to sustainable circular bioeconomy. Impact valuation methods [40] need to be mainstreamed in all business planning and reporting (in the context of SDG target 12.6.1), which means the boundary for measurement or assessment is extended to encompass much more than the economics of the business and some societal agreement can be reached about the values, morals, ethics, and preferences used for such measurements. The route to a solution for this challenge requires stakeholder agreed methods for natural capital accounting [41, 42] and ways to capture the multiple values (e.g., intrinsic, utility, relational, bequest) of ecosystem services [43] to address SDG target 15.9, to integrate ecosystems and biodiversity values into national and local planning, and to “implement and report on System of Environmental-Economic Accounting (SEEA) accounts” [44]. This will permit the mainstream prioritization of regulating and cultural ecosystem services over the satisfaction of demand and the development of systematic environmental information tracking to allow companies to understand the cycles and cascades necessary to develop biocircular products. Methods used at national scale such as the Circularity Gap reports [45], and the Eurostat Material Flow Analysis [46] can form the foundation for overcoming this challenge.
- *Challenge 3: Decoupling of economic growth from consumption and degradation of biological resources and natural capital associated with feedstock production*. Evidence is accumulating to suggest that bioeconomy does not cause this decoupling by default. For example, policy to promote biofuels has generated demand resulting in deforestation and conversion of crop systems that cause carbon and biodiversity impacts [47, 48]. Furthermore, some critical natural capital assets provide ecological functions and services that are poorly substitutable so their loss can never be justified [49]. Removal of subsidies for fossil fuel (SDG target 12.C) is necessary but does not go far enough. The route to a solution for this challenge requires (1) balancing the cost of biocircularized products relative to the subsidized fossil economy by factoring in the cost of ecosystem services and damage to natural capital for business-as-usual product and services; (2) mapping how economic growth can be driven by activity in circular loops rather than by raw material consumption; (3) designing innovative economic instruments that internalize the value of natural capital; and (4) preventing non-exchangeable capitals being used for offset measures.
- *Challenge 4: Integration across scales*. Four scales can be identified (i) the technical production of chemicals and materials; (ii) the technical production of products and objects; (ii) the operation of companies and sectors; and (iv) national and international trade, political, and social systems. For the principles of biocircularity to drive transition, integration is required over all four scales. Technical innovations at material or object scales will mean little unless companies develop biocircular business ecosystems, and these will not drive large scale transition unless integrated into global agreements. While biocircularity is well aligned with Sustainable Development Goal 12 (*Responsible consumption*), the route to a solution for this challenge should start with (1) using the principles of biocircularity to analyze new ideas from a very early stage so that new innovations (e.g., up to TRL 3) are not developed in isolation from the problems they will solve and the context of their deployment; (2) analyzing “solutions” in terms in the context of the continuum from feedstock to material to object to company to looping companies to national economy to global economy; (3) all solutions being analyzed from cradle (the biosphere) to cradle in the context of a verifiable value chain; and (4) developing and using new design tools created with biocircularity in mind.
- *Challenge 5*: *Fair access to and fair utilization of natural resources*. Natural capital is spatially specific but provides both local and global public goods, e.g., rainforests are home to indigenous communities and have a role in global hydrological cycles. The eco-ethical implications are significant [50], reflected in SDG target 15.6, and the Convention on Biological Diversity (CBD) [51] has been signed by all but four UN member states with a commitment to sustainable use of the components of biodiversity and the equitable sharing of the benefits derived from the use of genetic resources. CBD provides an established, international route to fair access considering the rights of many stakeholders but is not sufficient to achieve the targets of Sustainable Development Goal 10 (*Reduced inequalities*), to empower and promote inclusion for all and Sustainable Development Goal 15 (*Life on land*). This is perhaps the biggest challenge biocircularity faces because it requires an upheaval of how individuals, companies, governments, and society think about right of access and use of resources associated with land ownership. The route to a solution for this challenge should start with finding ways to incorporate pluralistic values and community values with business, economic, and governance frameworks. There is also a need to research ways of combining existing methods for holistic social assessment of circular economy [52].
- *Challenge 6: Management of renewable energy provisioning*. With current design and technology, the energy demand for reuse, recovery, and recycling of materials and objects is perhaps greater than the energy demand associated with using virgin raw materials. This issue has led to the suggestion that circular economy is akin to an unachievable perpetual motion machine [53]. This limitation will be reduced in proportion to the amount of carbon neutral renewable energy harnessed globally. Removal of subsidies for fossil fuel (target 12.C) is an essential step to meeting the challenge. The route to a solution for this challenge requires (1) design for minimum energy demand for circularity; (2) maximizing renewable energy supply perhaps with limited requirement for biofuels; and (3) the elimination of fossil fuels.
- *Challenge 7*: *A clear transition pathway to circular bioeconomy*. Transition theory lies at the intersection of political science, sociology, and management theory, with contributions from natural sciences, engineering, economics, and geography [54]. Theories emerge from historical transitions, success and failure stories, policy analysis, and analysis of social groups (e.g., business communities or grassroots movements). The problem faced by sustainability transition is that it must happen over the long term and a wide area, so short-term changes at specific locations do not seem to make much difference or may not even be seen as successful because the multi-dimensional nature of sustainability makes the likelihood of a failure quite possible. The route to a solution for this challenge will require transdisciplinary collaboration that addresses time, scale, scope, direction, system, and technology associated with the large socio-technical systems that enable urbanized, industrial societies to thrive. It will also require quantitative data and robust models on which to build decision-making. Two key issues need to be addressed by multi-disciplinary research groups supported by both public and private sector funds: (1) development of a biocircularity design ethos that can be integrated with all actors and stakeholders of the bioeconomy and (2) codesign of a number of situation specific transition pathways from early twenty-first century business-as-usual to a sustainable, circular bioeconomy prioritizing holistic system design, social justice, and restoration of natural capital.
- *Challenge 8*: *Integrating biocircularity with the food system*. Competition for space to balance food production whilst not depleting natural capital will need to be managed to enable a sustainable circular bioeconomy. While the problem is wicked [55], the high-level principles for constraining a solution are definable. A route to a solution for this challenge includes (i) finding and eating diets that minimize the land area needed to feed the global population a nutritious, healthy diet with minimum impact on natural capital (to meet the demands of SDG targets 2.1, 2.2, 2.4, 12.8, 12.C); (ii) estimating the prioritization of non-food feedstocks to provision society with its non-food needs (to meet the demands of SDG target 12.2); (iii) estimating the maximum provisioning from the food system within the limits of sustainable agriculture and minimization of wasted food (to meet SDG target 12.3); (iv) for the remaining land area and considering co-product streams from the food land area, estimating the maximum optimum provisioning that can be achieved within the limits of sustainable agriculture; (v) estimating global demand for non-food materials that can be provided by the bioeconomy; and (vi) calculating the amount of fossil-derived material currently in the technosphere that will have to pump-prime circularity in order for human society to be provisioned within the limits of end-of-life losses (related to SDG target 12.5) and sustainable production. Even at a crude level, these calculations would provide an estimate of the gap between current demand and sustainable consumption, which would indicate the degree to which biocircularity can enable sustainable society and how much behavior change will be required.

## Concluding Remarks

For sustainable, circular bioeconomy to work a balance is needed between the production capacity provided by the natural environment (the biogenic carbon feeding the circular bioeconomy [56]) and demand for materials, energy, and products. Ultimately, the amount of material in circulation has to buffer the difference between safe supply and current demand. If circular bioeconomy remains a poorly understood concept, with ill-defined limits, poorly defined measurements, a plethora of alternative assessment protocols, and few clear measures of success, it is susceptible to accusations of either greenwash or collapse of the ecosystem services relied on to support the economy before transitions are possible. At present, society lacks the data and means of analysis to manage transition to sustainable circular bioeconomy. Biocircularity provides a firm foundation for defining and measuring by considering sustainability in terms of natural capital from local to global scale and offering the basis for quantifying both circulations and impacts, rather than relying on one or more eco-efficiency metrics related to a specific function. The concept of biocircularity brings the challenges faced to achieve a sustainable, circular bioeconomy into sharp focus. Governance, policy, resources, research, and human effort should be devoted to achieving successful sustainable, circular bioeconomy by using biocircularity as the framework for measuring success. All stakeholders need to move towards a coherent understanding of what is needed to move society to a sustainable footing. To date, much of the scientific literature and commercial developments of circular bioeconomy have focused on waste valorization, i.e., processing feedstocks into useful materials or energy or simply displacing mineral feedstocks with biological feedstocks, regardless of whether either can be created or used sustainably. This approach is clearly not enough as it will not decouple production from consumption and impact and will not place inherently unsustainable production systems, such as the current food system, on a sustainable footing. The next step is to apply biocircularity to existing case studies and for the design of new systems. A bioeconomy that does not fully incorporate circularity will not offer a route to carbon neutrality, low impact, or maintenance and enhancement of natural capital. To find a route to a sustainable future, society needs to adopt a rigorous and realistic understanding of sustainable, circular bioeconomy, and the need to use quantitative tools to plan for and design reliable approaches to harnessing natural resources. Biocircularity offers a framework for design and measuring success that can be used by governments, policy makers, resource managers, researchers, and industry.

## Data Availability

Not applicable.
